# Supplementary material for: Structural Basis of PE_PGRS Polymorphism, a Tool for Functional Modulation
Source: Biomolecules. 2023 May 10;13(5):812. doi: 10.3390/biom13050812 (PMC10216338; doi:10.3390/biom13050812)
Supplement: Supplementary file 1 [file biomolecules-13-00812-s001.zip › biomolecules-2267335-supplementary.pdf]

Supplementary Table S1: List of the PE_PGRS33 protein variants and their association with the genetic polymorphisms as determined in a collection of 1024 Mtb clinical isolates.

Combination of sequence variation (according to Talarico 2007)(according to Kramarska 2021)(new code)	Variant	Number of clinical isolates: Talarico 2007	Basu 2007	Wang 2011	MCEvoy 2012	Camassa 2017	Gupta 2022	Summary	Percentage	Lineage 1	Lineage 2	Lineage 3	Lineage 4	Animal superlineage	Lineage 5	Lineage 6
wt	$_{a}$PGRS33^{allRv}	233		41	12	42	29	357	34,87	16	2	13	52			
I4	$_{b}$PGRS33^{all2}	167		5	13	41		226	22,08		2		52			
I4;D14;S4	$_{c}$PGRS33^{all26}	61		1		6	20	87	8,60	26						
I6	$_{d}$PGRS33^{all60}			44			43	87	8,60	13		23	7			
D10	PGRS33^{all3}	44						44	4,30							
I4	PGRS33^{all27}	29						29	2,83							
I4;D2;D14;S3;S4;S13	PGRS33^{all29}	7				11		18	1,76	11						
D5	PGRS33^{all4}	16						16	1,56							
I2	PGRS33^{all28}	15						15	1,46							
D24	PGRS33^{all56}				2	11		13	1,27				13			
I4;S4;S20	PGRS33^{all5}	11						11	1,07							
S14	PGRS33^{all6}	6				3		9	0,88				3			
I6;I8	$_{f}$PGRS33^{all82}						8	8	0,78			4				
ID1;S16	PGRS33^{all31}	6						6	0,59							
I4;S4;D19	PGRS33^{all47}					6		6	0,59			6				
I1;I5	PGRS33^{all7}	5						5	0,49							
D14	$_{g}$PGRS33^{all37}	3			1			4	0,40				1			
I1;I4;S4	PGRS33^{all22}	1	1			2		4	0,39				2			
I4;D11;D14;S4	PGRS33^{all32}	4						4	0,39							
I4;D5;D14;S4	PGRS33^{all33}	4						4	0,39							
D30; S20	PGRS33^{all70}			4				4	0,39							
S17	PGRS33^{all19}	1				2		3	0,29				2			

I4;S22;S4	PGRS33^{all45}					3	3	0,29				2		1
D1	PGRS33^{all9}	3					3	0,29						
I3;I4;S4	$_{h}$PGRS33^{all41}	2					2	0,20						
I4;D13;S4	PGRS33^{all10}	2					2	0,20						
D11	PGRS33^{all11}	2					2	0,20						
D17;S4	PGRS33^{all12}	2					2	0,20						
I4;D12;D14;S16	PGRS33^{all34}	2					2	0,20						
D9	PGRS33^{all35}	2					2	0,20						
I4;D26;S4	PGRS33^{all58}					2	2	0,20			2			
S29	PGRS33^{all66}			2			2	0,20						
D14	PGRS33^{all71}				2		2	0,20	2					
D19	PGRS33^{all73}				2		2	0,20		2				
I7	PGRS33^{all90}			1			1	0,10						
S10	PGRS33^{all16}	1					1	0,10						
S15	PGRS33^{all17}	1					1	0,10						
S12	PGRS33^{all18}	1					1	0,10						
I4;S1;S4;S11	PGRS33^{all21}	1					1	0,10						
I4;D7;S4	PGRS33^{all23}	1					1	0,10						
I4;S4;S5	PGRS33^{all24}	1					1	0,10						
D10;D18	PGRS33^{all25}	1					1	0,10						
I4;D14;S4;S21	PGRS33^{all36}	1					1	0,10						
D8	PGRS33^{all40}	1					1	0,10						
D6	PGRS33^{all43}	1					1	0,10						
D16;S14	PGRS33^{all44}	1					1	0,10						
D2;S4;I4	PGRS33^{all46}					1	1	0,10					1	
D20	PGRS33^{all48}		1				1	0,10						
D21	PGRS33^{all49}		1				1	0,10						
D22	PGRS33^{all50}		1				1	0,10						
D23*	PGRS33^{all51}		1				1	0,10						
ID2	PGRS33^{all52}		1				1	0,10						
D24;S23	PGRS33^{all53}					1	1	0,10			1			

S24	PGRS33^{all54}			1		1	0,10				1
D25	PGRS33^{all55}			1		1	0,10				1
I4;S4;S25	PGRS33^{all57}			1		1	0,10				1
D27;S4;S26	PGRS33^{all59}			1		1	0,10			1	
D29	PGRS33^{all63}	1				1	0,10				
S22	PGRS33^{all67}	1				1	0,10				
S30	PGRS33^{all68}	1				1	0,10				
D14; S32	PGRS33^{all72}		1			1	0,10	1			
D19;S31	PGRS33^{all74}		1			1	0,10			1	
D19;S26	PGRS33^{all75}		1			1	0,10			1	
D32	PGRS33^{all76}		1			1	0,10		1		
I4;S4;S34	PGRS33^{all77}		1			1	0,10				1
D14;S33	PGRS33^{all79}		1			1	0,10				1
D31	PGRS33^{all80}		1			1	0,10				1
ID1;I4;S4;S16	PGRS33^{all81}		1			1	0,10				1
I8	PGRS33^{all85}				1	1	0,10			1	
I10	PGRS33^{all87}				1	1	0,10			1	
I6;I12	PGRS33^{all89}				1	1	0,10	1			

We grouped "functionally" equivalent variant in a single variant as outlines below:

a: PGRS33^{allRv} includes variants coded by alleles with the sSNP S6 (n°=1), S7 (n°=1), S9 (n°=1), S27 (n°=1), S28 (n°=1) and S35 (n°=3);

b: PGRS33^{all2} includes variants coded by alleles with the sSNP S2-S4 (n°=5), S4 (n°=215), S18 (n°=1) or the I4 insertion;

c: PGRS33^{all26} includes variants coded by alleles with the sSNP S4-I4-D14 (n°=67), I6-D33 (n°=20) and the D28 (n°=1) indels;

d: PGRS33^{all60} includes variants coded by alleles with the sSNP S4 (n°=44) and I9-D33 (n°=1) indels in addition to the I6 insertion;

e: PGRS33^{all27} includes variants coded by alleles with the sSNP S4-D4 (n°=22) and S4-S19-D14 (n°=7)in addition to the I4 insertion;

f: PGRS33^{all82} includes variants coded by alleles with the I11 (n°=1) insertion in addition to the I6-I8 insertions;

g: PGRS33^{all60} includes variants coded by alleles with the sSNP S4-S8 (n°=1), S19 (n°=1) and S4-D15 (n°=1) in addition to the D14 deletion;

h: PGRS33^{all41} includes variants coded by alleles with the sSNP S4-I3-I4 (n°=1) and S4-I4-D3-D4 (n°=1);

References

Basu, S.; Pathak, S.K.; Banerjee, A.; Pathak, S.; Bhattacharyya, A.; Yang, Z.; Talarico, S.; Kundu, M.; Basu, J. Execution of Macrophage Apoptosis by PE_PGRS33 of Mycobacterium Tuberculosis Is Mediated by Toll-like Receptor 2-Dependent Release of Tumor Necrosis Factor-Alpha. *J. Biol. Chem.* **2007**, *282*, 1039–1050. https://doi.org/10.1074/jbc.M604379200.

Camassa, S.; Palucci, I.; Iantomasi, R.; Cubeddu, T.; Minerva, M.; De Maio, F.; Jouny, S.; Petruccioli, E.; Goletti, D.; Ria, F.; et al. Impact of Pe_pgrs33 Gene Polymorphisms on Mycobacterium Tuberculosis Infection and Pathogenesis. *Front Cell Infect. Microbiol.* **2017**, *7*, 137. https://doi.org/10.3389/fcimb.2017.00137.

Gupta, R.; Pandey, M.; Pandey, A.K.; Tiwari, P.K.; Amrathlal, R.S. Novel Genetic Polymorphisms Identified in the Clinical Isolates of Mycobacterium Tuberculosis PE_PGRS33 Gene Modulate Cytokines Expression and Promotes Survival in Macrophages. *J. Infect. Public Health* **2022**, *15*, 245–254. https://doi.org/10.1016/j.jiph.2022.01.001.

McEvoy, C.R.E.; Cloete, R.; Müller, B.; Schürch, A.C.; Helden, P.D.V.; Gagneux, S.; Warren, R.M.; Pittius, N.C.G. van Comparative Analysis of Mycobacterium Tuberculosis Pe and Ppe Genes Reveals High Sequence Variation and an Apparent Absence of Selective Constraints. *PLoS ONE* **2012**, *7*, e30593. https://doi.org/10.1371/journal.pone.0030593.

Talarico, S.; Zhang, L.; Marrs, C.F.; Foxman, B.; Cave, M.D.; Brennan, M.J.; Yang, Z. Mycobacterium Tuberculosis PE_PGRS16 and PE_PGRS26 Genetic Polymorphism among Clinical Isolates. *Tuberculosis* **2008**, *88*, 283–294. https://doi.org/10.1016/j.tube.2008.01.001.

Wang, J.; Huang, Y.; Zhang, A.; Zhu, C.; Yang, Z.; Xu, H. DNA polymorphism of Mycobacterium tuberculosis PE_PGRS33 gene among clinical isolates of pediatric TB patients and its associations with clinical presentation. Tuberculosis 2011, 91, 287–292, https://doi.org/10.1016/j.tube.2011.05.001.

Supplementary Table S2. List of the genetic polymorphisms of pe_pgrs33 gene of Mtb and identified as indicated by Talarico et al. (2008).

Variation nomenclature	Genomic position	Genomic change	Amino acid change
**Insertions**			
**I1**	597	+ 18 bp	*in frame* + 2 Gly-Gly-Ala at 199
**I2**	1039	+ 87 bp	*in frame* + 29 aa at 346
**I3**	1233	+ 270	*in frame* + 90 aa at 411
**I4**	1240	+ 9 bp	*in frame* + Gly-Gly-Ala at 414
**I5**	1409	+ 9 bp	*in frame* + 3 aa at 470
**I6**	1224	+ 9 bp	*in frame* + Ala-Gly-Gly at 408
**I7**	1076	+ 2 bp	*in frame* + Gly-Ser at 358
**I8**	377	+ 2 bp	*Frame shift change, STOP codon at 138*
**I9**	1051	+ 1 bp	*Frame shift change, STOP codon at 437*
**I10**	1232	+ 9 bp	*in frame* + Ala-Gly-Gly at 411
**I11**	588	+ 1 bp	*Frame shift change, STOP codon at 239*
**I12**	1108	+ 1 bp	*Frame shift change, STOP codon at 437*
**Deletions**			
**D1**	131-133	- 3 bp	*in frame* - Asp44
**D2**	416-487	- 72 bp	*in frame* - 4 Gly-Gly-X at 139
**D3**	421-435	- 15 bp	*in frame* - 5 aa (ΔAsn141-Gly145)
**D4**	550-639	- 90 bp	*in frame* - 30 aa (ΔGly183-Ile212)
**D5**	556-564	- 9 bp	*in frame* - Gly-Gly-Ala at 186
**D6**	585-728	- 144 bp	*in frame* - 48aa (ΔAla195-Gly242)
**D7**	591-608	- 18 bp	*in frame* - 2 Gly-Ala-Gly at 197
**D8**	640-780	- 141 bp	*in frame* - 47 aa (ΔGly214-Gly260)
**D9**	643-816	- 174 bp	*in frame* - 58 aa (ΔAla215-Gly272)
**D10**	651-659	- 9 bp	*in frame* - Gly-Ala-Gly at 217
**D11**	681-689	- 9 bp	*in frame* - Gly-Ala-Gly at 227
**D12**	709-981	- 273 bp	*in frame* - 91 aa (ΔGly236-Gly326)
**D13**	769-810	- 42 bp	*in frame* - 14 aa (ΔGly257-Thr270)

**D14**	1014	- 1 bp	*Frame shift change, STOP codon at 374*
**D15**	1081-1443	- 363 bp	*in frame* -121 aa (ΔThr361-Gly481)
**D16**	1114-1209	- 96 bp	*in frame* - 32 aa (ΔAla371-Thr402)
**D17**	1226-1234	- 9 bp	*in frame* - Gly-Ala-Gly at 408
**D18**	1398-1406	- 9 bp	*in frame* - Gly-Asn-Gly at 466
**D19**	772-813	- 42 bp	*in frame* - 14 aa (ΔGly258-Gly271)
**D20**	552-639	- 90 bp	*in frame* - 30 aa (ΔGly184-Gly213)
**D21**	711-981	- 273 bp	*in frame* - 91 aa (ΔLeu237-Gly327)
**D22**	1116-1209	- 96 bp	*in frame* - 32 aa (ΔGly372-Ala403)
**D23**	588-729	- 144 bp	*in frame* - 48aa (ΔAla196-Gly243)
**D24**	640-648	- 9 bp	*in frame* -1 Gly-Gly-Ala at 214
**D25**	1129-1137	- 9 bp	*in frame* -1 Gly-Gly-Ala at 410
**D26**	547-585	- 39 bp	*in frame* -3 Gly-Gly-Ala at 183
**D27**	772-819	- 27 bp	*in frame* -3 Gly-Gly-Ala at 258
**D28**	642-644	- 1 bp	*Frame shift change, STOP codon at 374*
**D29**	1134-1136	- 1 bp	*Frame shift change, STOP codon at 431*
**D30**	639-647	- 9 bp	*in frame* - Gly-Ala-Gly at 214
**D31**	684-728	- 45 bp	*in frame* - 15 aa (ΔAla228-Gly242)
**D32**	554-555	- 2 bp	*Frame shift change, STOP codon at 238*
**D33**	1008	- 1 bp	*Frame shift change, STOP codon at 374*
**Insertion and deletion**			
**ID1**	insertion at 709; 710–816 deleted<br>32bp insertion; 107bp deletion		
**ID2**	deletion of 107bp, position 710-816; insertion of 32bp in position 817		
**Synonymous SNPs**			
**S1**	48	c→t	
**S2**	81	c→t	

**S3**	207	g→a	
**S4**	717	t→c	
**S5**	837	t→c	
**S6**	1068	g→c	
**S7**	1191	c→a	
**S8**	1224	t→c	
**S9**	1467	g→a	
**S27**	156	g→a	
**S28**	633	t→a	
**S31**	285	g→a	
**S35**	1220	a→g	
**Non-synonymous SNPs**			
**S10**	28	g→a	Ala→Thr
**S11**	47	c→t	Thr→Ile
**S12**	196	a→c	Ser→Arg
**S13**	347	c→t	Pro→Leu
**S14**	529	t→g	Ser→Ala
**S15**	599	g→c	Gly→Ala
**S16**	701	t→c	Val→Ala
**S17**	820	g→a	Gly→Ser
**S18**	959	g→c	Gly→Ala
**S19**	1166	g→a	Gly→Asp
**S20**	1172	c→t	Thr→Ile
**S21**	1484	a→g	Asn→Ser
**S22**	697	g→a	Gly→Ser
**S23**	278	c→a	Ala→Asp
**S24**	665	g→a	Gly→Asp
**S25**	493,494	aa→gc	Asn→Ala
**S26**	235	g→a	Ala→Thr
**S29**	629	g→a	Gly→Glu

**S30**	1219	t→a	Phe→Ile
**S32**	320	c→t	Ala→Phe
**S33**	689	g→c	Gly→Ala
**S34**	1367	g→a	Gly→Asp

Data are obtained from the following references:

Basu, S.; Pathak, S.K.; Banerjee, A.; Pathak, S.; Bhattacharyya, A.; Yang, Z.; Talarico, S.; Kundu, M.; Basu, J. Execution of Macrophage Apoptosis by PE_PGRS33 of Mycobacterium Tuberculosis Is Mediated by Toll-like Receptor 2-Dependent Release of Tumor Necrosis Factor-Alpha. *J. Biol. Chem.* **2007**, *282*, 1039–1050. https://doi.org/10.1074/jbc.M604379200.

Camassa, S.; Palucci, I.; Iantomasi, R.; Cubeddu, T.; Minerva, M.; De Maio, F.; Jouny, S.; Petruccioli, E.; Goletti, D.; Ria, F.; et al. Impact of Pe_pgrs33 Gene Polymorphisms on Mycobacterium Tuberculosis Infection and Pathogenesis. *Front Cell Infect. Microbiol.* **2017**, *7*, 137. https://doi.org/10.3389/fcimb.2017.00137.

Gupta, R.; Pandey, M.; Pandey, A.K.; Tiwari, P.K.; Amrathlal, R.S. Novel Genetic Polymorphisms Identified in the Clinical Isolates of Mycobacterium Tuberculosis PE_PGRS33 Gene Modulate Cytokines Expression and Promotes Survival in Macrophages. *J. Infect. Public Health* **2022**, *15*, 245–254. https://doi.org/10.1016/j.jiph.2022.01.001.

McEvoy, C.R.E.; Cloete, R.; Müller, B.; Schürch, A.C.; Helden, P.D.V.; Gagneux, S.; Warren, R.M.; Pittius, N.C.G. van Comparative Analysis of Mycobacterium Tuberculosis Pe and Ppe Genes Reveals High Sequence Variation and an Apparent Absence of Selective Constraints. *PLoS ONE* **2012**, *7*, e30593. https://doi.org/10.1371/journal.pone.0030593.

Talarico, S.; Zhang, L.; Marrs, C.F.; Foxman, B.; Cave, M.D.; Brennan, M.J.; Yang, Z. Mycobacterium Tuberculosis PE_PGRS16 and PE_PGRS26 Genetic Polymorphism among Clinical Isolates. *Tuberculosis* **2008**, *88*, 283–294. https://doi.org/10.1016/j.tube.2008.01.001.

Wang, J.; Huang, Y.; Zhang, A.; Zhu, C.; Yang, Z.; Xu, H. DNA polymorphism of Mycobacterium tuberculosis PE_PGRS33 gene among clinical isolates of pediatric TB patients and its associations with clinical presentation. *Tuberculosis* **2011**, *91*, 287–292, https://doi.org/10.1016/j.tube.2011.05.001.
